# Gut microbial ecology of the Critically Endangered Fijian crested iguana (*Brachylophus vitiensis*): Effects of captivity status and host reintroduction on endogenous microbiomes

**DOI:** 10.1002/ece3.7373

**Published:** 2021-03-26

**Authors:** Samuel J. Eliades, Joseph C. Brown, Timothy J. Colston, Robert N. Fisher, Jone B. Niukula, Kim Gray, Jhabar Vadada, Sia Rasalato, Cameron D. Siler

**Affiliations:** ^1^ Sam Noble Oklahoma Museum of Natural History and Department of Biology University of Oklahoma Norman Oklahoma USA; ^2^ Hope Zoo Preservation Foundation Kingston Jamaica; ^3^ Department of Biology University of Florida Gainesville Florida USA; ^4^ Western Ecological Research Center U.S. Geological Survey San Diego California USA; ^5^ National Trust of Fiji Islands Suva Fiji; ^6^ San Diego Zoo Wildlife Alliance San Diego California USA; ^7^ Ahura Resorts Suva Fiji

**Keywords:** conservation, headstart, husbandry, microbial restructuring, reptiles, wildlife management

## Abstract

Animals often exhibit distinct microbial communities when maintained in captivity as compared to when in the wild. Such differentiation may be significant in headstart and reintroduction programs where individuals spend some time in captivity before release into native habitats. Using 16S rRNA gene sequencing, we (i) assessed differences in gut microbial communities between captive and wild Fijian crested iguanas (*Brachylophus vitiensis*) and (ii) resampled gut microbiota in captive iguanas released onto a native island to monitor microbiome restructuring in the wild. We used both cloacal swabs and fecal samples to further increase our understanding of gut microbial ecology in this IUCN Critically Endangered species. We found significant differentiation in gut microbial community composition and structure between captive and wild iguanas in both sampling schemes. Approximately two months postrelease, microbial communities in cloacal samples from formerly captive iguanas closely resembled wild counterparts. Interestingly, microbial communities in fecal samples from these individuals remained significantly distinct from wild conspecifics. Our results indicate that captive upbringings can lead to differences in microbial assemblages in headstart iguanas as compared to wild individuals even after host reintroduction into native conditions. This investigation highlights the necessity of continuous monitoring of reintroduced animals in the wild to ensure successful acclimatization and release.

## Graphical abstract

We show that captivity impacts gut microbial assemblages in headstart animals compared with wild individuals long after host reintroduction into native conditions. This advances our understanding of microbiome diversity and evolution in a Critically Endangered reptile and shows how captivity influences the microbiome of reptiles in general.

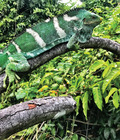

## 1 INTRODUCTION

Gastrointestinal microbial communities are critical to host health, contributing to an array of functions that impact host fitness and reproductive success such as nutrient acquisition based on digestive efficiency, hormone balance, and immune response (Cho & Blaser, 2012; Colston & Jackson, 2016; Fraune & Bosch, 2010; Ley et al., 2008). Given that gut microbiota serve essential roles in maintaining host well‐being, the study of these communities is a novel tool for wildlife conservation initiatives, particularly in programs involving ex situ animal care (Bahrndorff et al., 2016; Jiménez & Sommer, 2017; Redford et al., 2012; West et al., 2019). With few exceptions, a variety of species housed in captivity show disparate gut microbiomes compared with wild counterparts which may be caused by dietary differences, antibiotic treatments, exposure to other species in captivity, or various other potential drivers that alter microbial compositions (Alfano et al., 2015; Cheng et al., 2015; Clayton et al., 2016; Eigeland et al., 2012; McKenzie et al., 2017; West et al., 2019; Zhu et al., 2011). Such differences may be signs of dysbiosis, or perturbations of microbial communities that hinder system function and are often associated with negative health outcomes in hosts (Gilbert et al., 2016; West et al., 2019). For example, captivity has been linked to increases in potential pathogens within gastrointestinal microbial communities in mammals (Amato et al., 2016; Cheng et al., 2015; Wan et al., 2016; Wasimuddin et al., 2017), birds (Xie et al., 2016), and reptiles (Jiang et al., 2017; Kohl et al., 2017). Distinct gut microbiota between captive and wild hosts is especially significant in headstart and reintroduction conservation programs, as altered microbial communities or introduced pathogens in captive animals slated for release could hinder reintroduction success and survivorship in the wild due to reduced dietary efficiency or compromised immune response affecting survivorship (Bahrndorff et al., 2016; Jiménez & Sommer, 2017; Redford et al., 2012; West et al., 2019).

Headstart programs have become increasingly common management strategies to supplement declining wildlife populations at risk of extinction (McGowan et al., 2017; Redford et al., 2011; Tear et al., 1993). In these programs, young animals are reared in captivity past their most vulnerable life stages before being released to reinforce wild populations (Alberts, 2007; Ferguson et al., 1982). Historically, however, effective reintroduction of captive animals into the wild has been rare, with as few as 13% of such projects being deemed successful (Fischer & Lindenmayer, 2000; Mathews et al., 2005). Multiple factors have been linked to animal headstart and reintroduction difficulties including individual animal behavior (Alberts, 2007; Mathews et al., 2005) and ill‐suited release sites (Pérez‐Buitrago et al., 2008). More recently, microbial incompatibilities also have been suggested as possible impediments to reintroduction success (Bahrndorff et al., 2016; Jiménez & Sommer, 2017; Redford et al., 2012; West et al., 2019). However, no studies to date have examined gut microbiota in reintroduced species both pre‐ and postrelease to analyze microbial composition and acclimation of these communities to native habitats. Improved understanding of host natural microbiomes and microbial shifts associated with captivity and headstart animal release could help management practitioners to better prepare animals for reintroduction and increase headstart success of imperiled species.

The Fijian crested iguana (*Brachylophus vitiensis*) is an herbivorous lizard species endemic to dry and littoral forests in western Fiji (Fisher et al., 2019; Harlow, Fisher, & Grant, 2012). Since the species’ discovery in 1981, it has experienced sharp population declines throughout most of its limited range due to habitat loss and introduced predators (Fisher et al., 2019; Gibbons, 1981; Harlow et al., 2007). The Fijian crested iguana is listed on CITES Appendix S1 and as Critically Endangered by the IUCN Red List (Fisher et al., 2019; Harlow et al., 2012). To ensure the long‐term viability of this species in Fiji, a captive breeding and headstart program was established in 2010 with a specific focus on animals from the uninhabited island of Monuriki (Chand et al., 2016; Fisher et al., 2019). Monuriki Island crested iguanas are genetically distinct from all other crested iguana populations (Keogh et al., 2008), and the 2008 Iguana Species Recovery Plan prioritized Monuriki as the single most important site for immediate conservation action for this taxon (Fisher et al., 2019; Harlow et al., 2008). From 2010 to 2012, 20 adult iguanas were caught in the wild from Monuriki Island and transported to Kula Eco Park on the large island of Viti Levu to develop a captive breeding colony (Chand et al., 2016). Over the next six years, these 20 wild‐caught individuals were successfully bred in managed care at Kula Eco Park with the intention of headstarting and returning the offspring to their source island of Monuriki (Chand et al., 2016; Fisher et al., 2019). In mid‐May 2015, 32 captive‐bred crested iguanas were released onto Monuriki Island, with an additional 32 captive‐bred iguanas and 16 of the original adult wild founder iguanas released onto Monuriki in February 2017.

In 2017, we completed extensive sampling of gut microbial communities from Fijian crested iguanas in captivity at Kula Eco Park, wild iguanas on Monuriki Island, and previously captive iguanas released onto Monuriki to better understand how endogenous microbiomes are influenced by both human care and host reintroduction. In this study, we not only compare gut microbiomes in captive and wild lizards of a Critically Endangered species, but also assess the restructuring of microbiota in headstart animals reintroduced into native habitats. Additionally, by inventorying gut microbiota in Fijian crested iguanas using two sampling techniques, cloacal swabs and fecal samples, we address how sampling regime influences microbial data recovered and subsequent downstream analyses. While gut microbial diversity reported from cloacal and fecal sampling is often similar, significant discrepancies in relative abundances of microbial taxa between sampling types are well noted (Colston et al., 2015; Kohl et al., 2017; Stanley et al., 2015). We used both techniques to maximize our understanding of gut microbial ecology in *B. vitiensis* and to mitigate potential shortcomings associated with employment of a single sampling technique (Colston et al., 2015; Ren et al., 2016). The Fijian crested iguana headstart initiative represents a unique opportunity to address two important research questions: (i) How does captivity status effect the diversity and structure of gut microbiomes? and (ii) How do such communities respond to host reintroduction into native habitats? The results of this study have direct implications for the management and conservation of this Critically Endangered reptile species and for headstart and reintroduction programs globally.

## 2 MATERIALS AND METHODS

### 2.1 Animal maintenance and sample collection

**FIGURE 1:**
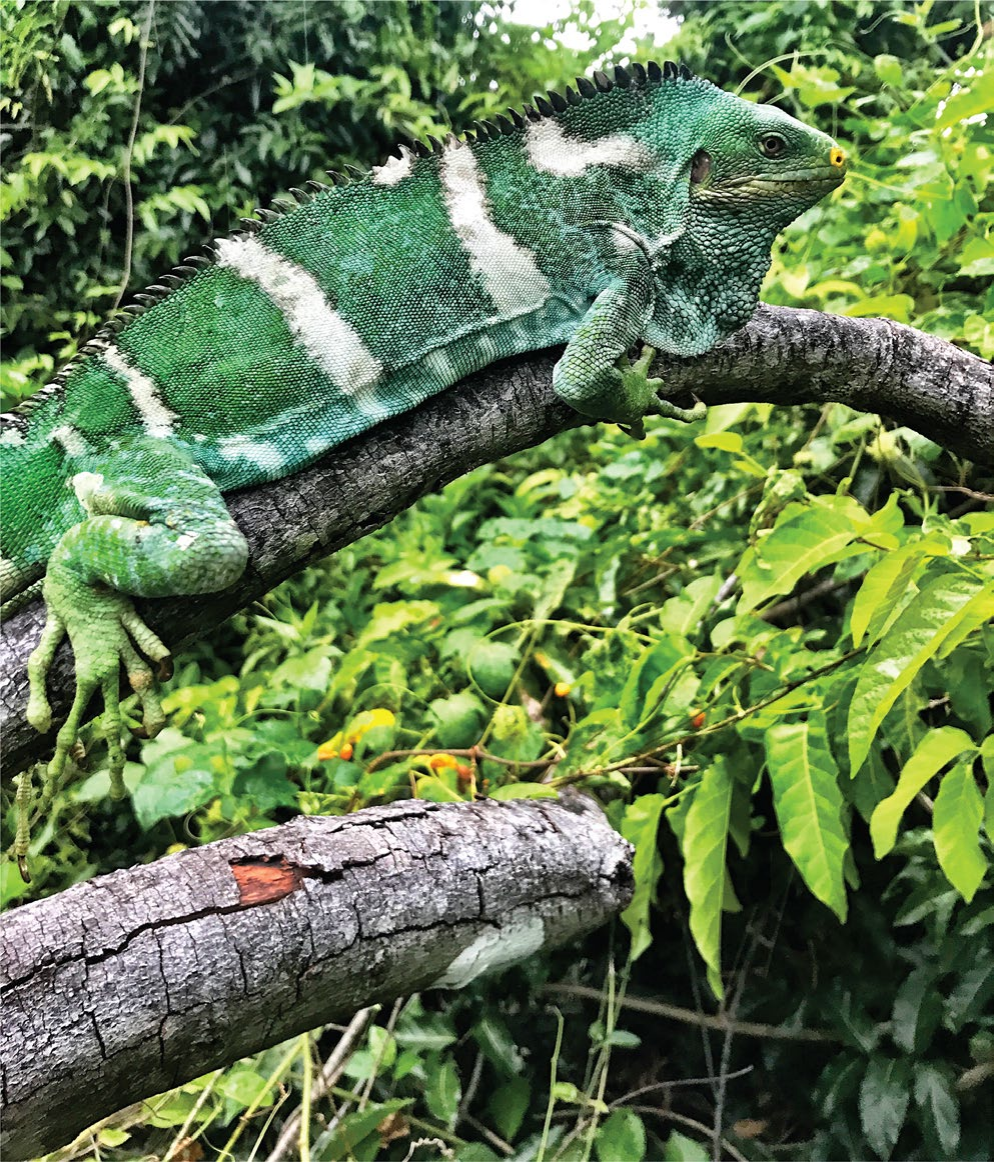
Adult Fijian crested iguana (*B. vitiensis*) perched in native habitat on Monuriki Island (Photograph by J.C.B.)

Located 45 km northwest of the main Fijian island of Vitu Levu, Monuriki Island (17°37'S, 177°02'E) is a small (45 ha, 216 m ele.), uninhabited island belonging to the Mamanuca Island group in western Fiji (Figure 1). From 2010 to 2012, 10 male and 10 female adult Monuriki Island crested iguanas were harvested from the wild and brought to Kula Eco Park on Viti Levu to initiate a captive breeding headstart program. These 20 wild‐caught crested iguanas were maintained at Kula Eco Park in a private facility specifically built for captive breeding of Monuriki crested iguanas. Iguana cages were made from galvanized steel and mesh, measuring 92 cm tall and 92 cm wide, with wood branches for arboreal perching. Iguanas were maintained on a daily diet of fresh salad made from local mixed greens and fruits. Adult iguanas were housed in pairs, while all captive‐bred offspring were kept in small groups of two to four individuals per cage. Nest boxes were placed in cages for gravid females. Once eggs were deposited by a female, they were immediately removed and placed in a separate incubator until hatching. Hatchlings were fed in the same manner as adults and juveniles, but salads were cut into smaller pieces. We implanted unique passive integrated transponder (PIT) tags subcutaneously into all iguanas for identification in the wild.

We collected samples from crested iguanas of four distinct life history groups: The original wild‐caught adult founder iguanas from Monuriki brought to Kula Eco Park for captive breeding (wild‐caught founders; WCF) from 2010 to 2012, captive‐born individuals released onto Monuriki in 2015 (CB2015), captive‐born individuals released onto Monuriki in 2017 (CB2017), and wild individuals on Monuriki (Wild). Further, we sampled microbiota in WCF and CB2017 individuals while in captivity and approximately 2 months after relocation onto Monuriki Island.

From 22 to 24 February 2017, we inventoried gut microbiota in WCF and CB2017 Fijian crested iguanas at Kula Eco Park using two sampling techniques, cloacal swabs and fecal samples. To collect cloacal samples, sterile, rayon‐tipped swabs were inserted approximately 3 cm into the cloacal opening of each animal and rotated 10 times. For fecal sample collection, iguanas were placed in individual prewashed pillowcases overnight and feces were retrieved opportunistically within 4–8 hr. Pillowcases were washed subsequent to each use. For efficient preservation of DNA in both sample types, swabs and fecal samples were placed into individual screw‐cap 1.5 ml cryovials with 750 µl Xpedition^TM^ Lysis/Stabilization Solution. These vials were subsequently inserted into a custom 3D‐printed plastic sleeve to hold the vials, bolted to a reciprocating saw attachment, inserted into a Milwaukee M12 Hackzall battery‐operated reciprocating saw, and shaken vigorously for 5 min to act as a mechanical homogenization device. Samples were stored at ambient temperature while in the field before transportation to the Sam Noble Oklahoma Museum of Natural History for curation and storage.

On 24 February 2017, we transported 16 WCF and 32 CB2017 (aged 12–28 months) iguanas from Kula Eco Park to Monuriki Island for assimilation into their source population. From the time of release to mid‐July 2017, we conducted standard night surveys for *Brachylophus* (Harlow et al., 2007) on Monuriki Island to monitor iguanas and sample gut microbial communities in the wild. Once iguanas were captured, the presence of a PIT tag allowed us to determine whether the individual was a WCF, CB2017, or CB2015 iguana, while all iguanas lacking PIT tags were classified as Wild individuals. Gut microbial samples were collected using the same methodologies as for iguanas in captivity at Kula Eco Park.

### 2.2 Microbial inventories

We extracted total DNA from 94 samples (52 cloacal and 42 fecal) from 39 host lizards using Zymo Quick‐DNA Fecal/Soil Microbe Kits. Both cloacal swabs and fecal samples were incubated at 65°C for 15 min on a dry heating block and then vortexed for 15 min on an Eppendorf ThermoMixer® at 23°C and maximum speed (2000 rpm) immediately prior to beginning Zymo's recommended protocol. We amplified the V4 region of the 16S rRNA gene using the index primers and PCR protocols of Kozich et al., (2013). PCR products were cleaned, normalized, and pooled using a SequelPrep Normalization Plate Kit (Invitrogen). Pooled libraries were purified using Agencourt® AMPure® magnetic bead capture and sent to the University of Oklahoma's Consolidated Core Lab (CCL) for sequencing using 515F and 806R primers targeting 2x300bp reads on an Illumina MiSeq sequencing platform (Caporaso et al., 2012).

Raw sequences were first paired and trimmed using AdapterRemoval2 v2.2.2 with default parameters (Lindgreen, 2012; Schubert et al., 2016). Cleaned sequences were clustered de novo into operational taxonomic units (OTUs) using UPARSE in USEARCH v11.0.667 at a minimum sequence identity of 97% and a minimum abundance of four (Edgar, 2013). Remaining sample curation and analysis were carried out in QIIME v1.9.1 (Caporaso et al., 2010). Taxonomies were assigned to OTUs using GreenGenes v13.8 (DeSantis et al., 2006). Archaea, chloroplast, mitochondria, PhiX, and other nonbacterial sequences were removed from processed OTU tables to ensure only bacterial sequences were included in downstream analyses. All 16S rRNA sequences have been deposited in the Sequence Read Archive (SRA) under accession no. PRJNA702127.

Among all samples (*n* = 94), a number were either duplicates (i.e., multiple subsamples of a single fecal deposit or cloacal swabs collected from the same host consecutively) or failed to generate sufficient sequencing coverage to produce meaningful microbial assessments. In instances where duplicate samples existed (*n* = 9), we retained only the sample with the greater sequencing depth. Of the remaining samples, those with fewer than 500 sequences (*n* = 2) were also removed to maximize sample inclusion against OTU coverage. The finalized dataset used for all subsequent analyses consisted of 83 samples (46 cloacal and 37 fecal) from 38 Fijian crested iguanas (Appendix S1). Within these datasets, five Fijian crested iguana hosts had complete time‐series sets (pre‐ and postrelease sampling) via cloacal swabbing and five had them through fecal sampling. Three individuals occurred in both groups and had complete sampling sets from the two methodologies (Appendix S1).

Rarefaction depths varied by comparison based on Good's coverage estimates (Good, 1953) and rarefaction curves to maximize sample inclusion against OTU coverage (Figure S1). For analyses inclusive of all samples and of cloacal samples exclusively, we rarefied to 500 reads per sample (Good's estimate all samples = 0.92 ± 0.03, range: 0.86–0.99; cloacal samples = 0.94 ± 0.03, range: 0.87–0.98). In analyses involving fecal samples exclusively, we rarefied to 3,350 sequences per sample (Good's estimate fecal samples = 0.98 ± 0.005, range: 0.97–0.99).

We compared a variety of community membership metrics across samples from Fijian crested iguana hosts. For all comparisons, we first calculated alpha‐diversity measurements including number of observed OTUs, the Shannon index (Shannon, 1948), and Faith's phylogenetic diversity (Faith's PD; Faith, 1992). Alpha‐diversity measurements were compared using analysis of variance (ANOVA) tests in R v3.6.2 (R Core Team, 2013) with the Tukey test used for post hoc analyses. The Kruskal–Wallis tests with Bonferroni's corrections were used in QIIME to compare relative abundances of bacterial taxa between treatment groups. In examining specific OTUs, BLAST (Altschul et al., 1990) was used to compare novel sequences against those available in the National Center for Biotechnology Information's (NCBI) nucleotide database.

Community diversity and structure were compared using principal coordinates analysis (PCoA) on beta‐diversity metrics including weighted and unweighted UniFrac distances (Lozupone & Knight, 2005) and the binary Jaccard index (Jaccard, 1901). Beta‐diversity matrices and PCoA plots were generated from the same rarefied datasets used to measure alpha‐diversity metrics. The adonis function in the vegan v2.3_4 package (Oksanen et al., 2016) of R v3.3.1 (R Core Team, 2013) was used on beta‐diversity distance matrices with 999 permutations to compare community composition between groups statistically.

### 2.3 Sample comparisons

We first analyzed bacterial composition across all 83 samples (Appendix S1) and then split the dataset into cloacal and fecal subsets to examine general patterns between sample types. Following broad overviews of the data, we tested the effects of captivity status on gut bacterial communities in crested iguana hosts and examined for microbial restructuring in reintroduced lizards postrelease.

To determine the influences of captivity status on gut microbial communities, we used snapshot analyses of cloacal and fecal samples taken from WCF, CB2017, CB2015, and Wild lizards. For cloacal comparisons, we included 35 samples collected between 22 February and 2 March 2017 (Appendix S1). This subset included 10 WCF, 13 CB2017, three CB2015, and nine Wild individuals. In our subsequent fecal analyses, we included 26 fecal samples collected between 22 February and 1 March 2017 (Appendix S1). This dataset encompassed fecal samples from nine WCF, nine CB2017, two CB2015, and six Wild iguanas. In addition to comparing microbial communities across four treatments, we also ran all analyses between just two conditions, captive (WCF and CB2017 grouped) and noncaptive (CB2015 and Wild grouped) (Ren et al., 2016).

We sought to assess the effects of release on lizard microbiota using both cloacal and fecal samples collected roughly 2 months after host reintroduction to Monuriki. We collected cloacal samples from five recently released lizards, one WCF and four CB2017, between 24 April and 11 May 2017 (Appendix S1). We compared microbial communities from these samples against those in the initial 23 captive animal cloacal samples (10 WCF, 13 CB2017) and the initial 12 noncaptive samples (nine Wild, three CB2015). We also compared six novel fecal samples (one WCF, five CB2017) collected between 2 and 17 May (Appendix S1) against the 18 initial captive fecal samples (nine WCF, nine CB2017) and eight noncaptive fecal samples (two CB2015, six Wild). In both instances, we sought to determine whether gut microbiomes were more similar to captive communities or noncaptive communities two months after host reintroduction.

## 3 RESULTS

### 3.1 General patterns in Fijian crested iguana microbiota

Our curated dataset of 83 samples generated 898,625 reads with a minimum read depth of 540, a maximum of 30,503, and a median of 9,883 reads per sample. Among the 46 cloacal samples only, 410,545 reads were recovered with a minimum read depth of 540 sequences per sample, maximum of 25,304, and median read depth of 8,521.5. The 37 fecal samples produced 488,080 reads with a minimum, maximum, and median read depth of 3,378, 30,503, and 12,558 reads per sample, respectively.

Fijian crested iguana microbiome samples averaged 85 unique OTUs per 500 reads, the Shannon index varied from 0.93 to 6.32 (mean = 4.77 ± 1.28), and Faith's PD varied from 2.76 to 14.33 (mean = 9.34 ± 2.87). The average Jaccard distance between pairs of samples was 0.83 suggesting that any two samples shared ~17% of their OTUs on average. Across rarefied sequences, six OTUs were found in ≥ 70% of all samples, one *Oscillospira* sp., one *Phascolarctobacterium* sp., two unidentified taxa in the family Enterobacteriaceae, and two unidentified taxa in the families Clostridiaceae and Lachnospiraceae. At a rarefied depth of 500 reads per sample, most sequences (91.8%) belonged to four phyla: Firmicutes (48.3%), Proteobacteria (18.4%), Actinobacteria (13.9%), and Bacteroidetes (11.1%).

The average number of OTUs per cloacal sample was 68 (sequence depth = 500 rarified reads/sample), the Shannon index varied from 0.81 to 6.07 (mean = 4.09 ± 1.32), and Faith's PD varied from 2.8 to 12.66 (mean 7.88 ± 3.04). Jaccard distances averaged 0.86 across pairs of cloacal samples, a slight increase when compared to that among all samples. Just four OTUs were identified in ≥ 70% rarefied cloacal sequences, one *Corynebacterium* sp., an unidentified microbe in Clostridiaceae, and two unidentified taxa in Enterobacteriaceae. The majority of cloacal reads (95.1%) belonged to the same four dominant phyla as in all samples: Firmicutes (37.2%), Proteobacteria (27.7%), Actinobacteria (24.3%), and Bacteroidetes (5.9%).

Within fecal samples and at a sequencing depth of 3,350 quality‐controlled reads, the average number of OTUs found was 224, the Shannon index varied from 5.14 to 6.63 (mean = 5.90 ± 0.37), and Faith's PD varied from 11.94 to 21.69 (mean = 17.13 ± 2.05). The average Jaccard distance between any pair of fecal samples was 0.65, suggesting more similarity among fecal samples compared with among cloacal samples. Across all fecal samples, 90 OTUs were found in ≥ 70% of samples and seven OTUs were found in 100% of fecal samples. These included three *Bacteroides* spp., one *Parabacteroides* sp., an unidentified taxon in Lachnospiraceae, one in Enterobacteriaceae, and a third in Ruminococcaceae. Most rarefied reads (86.6%) belonged to just three phyla: Firmicutes (61.5%), Bacteroidetes (18.1%), and Proteobacteria (7.0%), while Actinobacteria comprised only 0.8% of rarified fecal reads.

### 3.2 Comparison of microbiota in captive and noncaptive iguanas via cloacal samples

**FIGURE 2:**
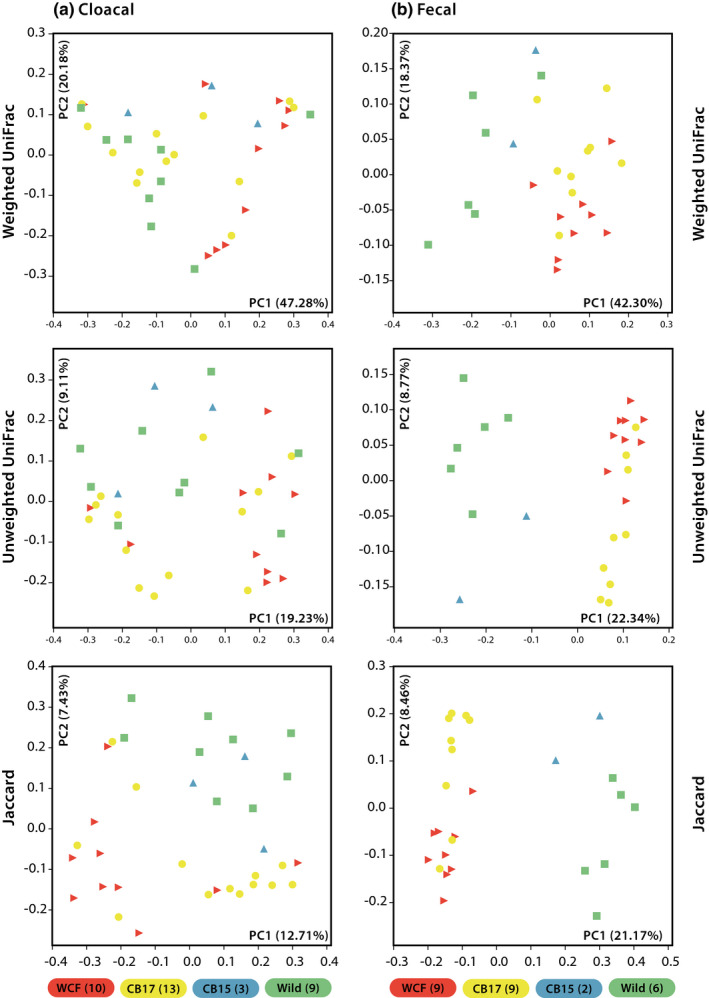
Principal coordinates analysis plots of initial (2017) (a) cloacal swabs and (b) fecal samples across four Fijian crested iguana treatment groups. Treatment groups include wild‐caught founders (WCF) in captivity and captive‐born headstart individuals (CB2017) in captivity at Kula Eco Park on Viti Levu, Fiji, as well as captive‐born individuals released onto Monuriki Island in 2015 (CB2015) and fully wild individuals on Monuriki Island (Wild). The number of individual samples per treatment group is indicated in parentheses

Comparisons of cloacal samples from Fijian crested iguanas of treatment groups WCF, CB2017, CB2015, and Wild yielded no significant differences in measured alpha‐diversity metrics (Figure S2). This lack of differentiation remained even when samples were grouped as captive (WCF and CB2017 grouped) and noncaptive (CB2015 and Wild grouped) treatments (Figure S2). PCoA plots of beta‐diversity metrics showed limited clustering when grouping both by four treatments and by captive and noncaptive lizards (Figure 2a). Among all four treatments, adonis tests determined significant differentiation in unweighted UniFrac distances (*R* ^2^ = 0.1412, *p* = 0.004) and Jaccard distances (*R* ^2^ = 0.1445, *p* = 0.001), while weighted UniFrac distances (*R* ^2^ = 0.1448, *p *= 0.075) were not significantly distinct. Grouping by captive and noncaptive types produced similar, yet weaker, results in unweighted UniFrac distances (*R* ^2^ = 0.0641, *p* = 0.007), Jaccard distances (*R* ^2^ = 0.0646, *p* = 0.002), and weighted UniFrac distances (*R* ^2^ = 0.0460, *p* = 0.174). The average Jaccard distance between pairs of cloacal samples in this subset was 0.85 and remained similar within treatment groups (WCF = 0.81, CB2017 = 0.82, CB2015 = 0.75, Wild = 0.88; captive = 0.83, noncaptive = 0.86).

Rarefied cloacal samples across all groups in this subset were dominated by Firmicutes (37.7%), Proteobacteria (26.2%), Actinobacteria (24.9%), and Bacteroidetes (6.5%) with some differentiation among treatments (Figure S3). At 500 sequences per sample, the Kruskal–Wallis tests identified two OTUs that varied significantly in relative abundance between all four treatments following Bonferroni's corrections. These included one *Cupriavidus* sp. (WCF mean reads = 0, CB2017 = 0, CB2015 = 0.7, Wild = 0) and an unidentified taxon in Coriobacteriaceae (WCF mean reads = 0, CB2017 = 0, CB2015 = 2.0, Wild = 0). Both of these differentiations are likely due to limited sampling in the CB2015 category (*n* = 3). When comparing captive and noncaptive samples, one OTU, an unidentified taxon in Micrococcaceae, was found to differ between treatment groups (mean captive reads = 19.1, noncaptive = 0). BLAST queries of this specific sequence returned a 99.6% match to *Nesterenkonia* sp. strain MadaFrogSkinBac.DB‐0.3605. While not significantly distinct between treatments, a number of OTUs were present in rarefied captive samples that were absent in noncaptive ones (Appendix S2). Notably, these included another *Nesterenkonia* sp. (captive mean reads = 37.7), one *Brevibacterium* sp. (captive mean reads = 12.5), and one *Brachybacterium* sp. (captive mean reads = 11.2).

### 3.3 Comparison of microbiota in captive and noncaptive iguanas via fecal samples

**FIGURE 3:**
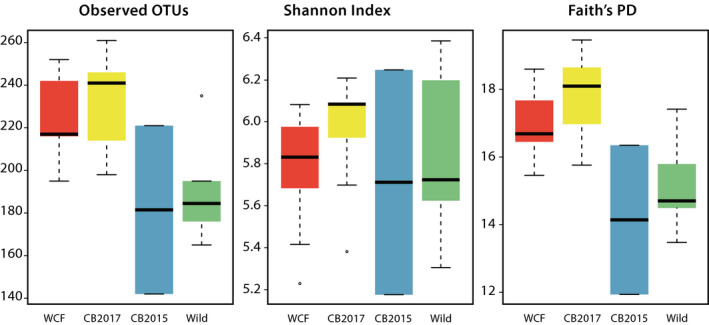
Alpha‐diversity metrics of initial (2017) fecal samples across four treatment groups. Treatments included wild‐caught founder (WCF) iguanas in captivity, captive‐born headstart individuals (CB2017) in captivity, captive‐born individuals released onto Monuriki in 2015 (CB2015), and fully wild individuals on Monuriki Island (Wild). Paired symbols denote significantly distinct treatment groups

We found significant differences in the number of OTUs (*p* = 0.005; WCF = 223, CB2017 = 233, CB2015 = 181.5, Wild = 190) and in Faith's PD (*p* = 0.001; WCF = 17.0, CB2017 = 17.8, CB2015 = 14.1, Wild = 15.1) but not in the Shannon index when comparing fecal samples across all four treatments (Figure 3). Post hoc analyses of observed OTUs found significant differentiation between CB2017 and Wild samples (*p* = 0.012), while remaining comparisons were insignificant. Post hoc analyses of Faith's PD results revealed significant differentiation between CB2017 and CB2015 (*p* = 0.011) and CB2017 and Wild (*p* = 0.005) treatments. Remaining pairwise comparisons were insignificant. Grouping by captive and noncaptive statuses again resulted in significant differences in the number observed OTUs (*p* < 0.001; captive mean = 229, noncaptive mean = 188) and in Faith's PD (*p* < 0.001, captive mean = 17.4, noncaptive mean = 14.9) but not in the Shannon index (Figure S4). PCoA plots showed evident clustering among all four treatments and when grouped as captive and noncaptive samples (Figure 2b). Adonis analyses showed significant differences between the four conditions in weighted UniFrac distances (*R* ^2^ = 0.4297, *p* = 0.001), unweighted UniFrac distances (*R* ^2^ = 0.3302, *p* = 0.001), and Jaccard distances (*R* ^2^ = 0.3142, *p* = 0.001). Captive and noncaptive comparisons showed similarly significant yet slightly weaker results in weighted UniFrac (*R^2^* = 0.3162, *p* = 0.001), unweighted UniFrac (*R^2^* = 0.2122, *p* = 0.001), and Jaccard distances (*R^2^* = 0.2036, *p* = 0.001). Pairs of fecal samples averaged a Jaccard distance of 0.65 with some deviation within treatment groups (WCF = 0.57, CB2017 = 0.55, CB2015 = 0.57, Wild = 0.63; captive = 0.57, noncaptive = 0.64).

The most prevalent phyla among rarefied fecal reads included Firmicutes (66.5%), Bacteroidetes (16.1%), and Proteobacteria (6.5%) contributing to 89.2% of sequences. Synergistetes (2.4%), Planctomycetes (2.3%), Tenericutes (2.0%), and Verrucomicrobia (1.9%) also contributed to general relative diversity present while Actinobacteria accounted for just 0.7% of rarefied reads (Figure S5). The Kruskal–Wallis tests identified one OTU that varied in abundance across all four groups, an unidentified Clostridiales (WCF mean reads = 0, CB2017 = 0, CB2015 = 1.5, Wild = 0) though significance of this difference is likely due to limited sampling of CB2015 individuals (*n* = 2) in this subset. Comparisons of captive and noncaptive microbial communities from crested iguana fecal samples identified seven OTUs that varied significantly between treatments. Three of these OTUs, one *Coprococcus* sp. (captive mean = 0.3, noncaptive = 26.5), an unidentified Coriobacteriaceae (captive mean = 0, noncaptive = 3.9), and an unidentified Mogibacteriaceae (captive mean = 0, noncaptive = 4.3), were more prevalent in noncaptive animals than in captive ones (Appendix S2). The remaining four OTUs were common in rarefied captive animal communities but absent from noncaptive counterparts. These OTUs included one *Ruminococcus* sp. (captive mean = 95.3, noncaptive = 0), an *Acetobacterium* sp. (captive mean = 82, noncaptive = 0), an unidentified Christensenellaceae (captive mean = 53.4, noncaptive = 0), and one *Bacteroides* sp. (captive mean = 25.3, noncaptive = 0; Appendix S2). References of the unidentified Christensenellaceae sequence against published data in BLAST returned hits only to uncultured bacterial clones. A litany of additional OTUs were present in rarefied captive fecal samples that were not recovered in noncaptive ones (Appendix S2). Among these included an unidentified taxon in Synergistaceae (captive mean = 116.9), two unidentified Christensenellaceae (captive means = 58.6, 38.9), one *Akkermansia* sp. (captive mean = 29.3), another *Ruminococcus* sp. (captive mean = 18.7), an unidentified Clostridiales (captive mean = 15.1), and one *Coprococcus* sp. (captive mean = 11.0). BLAST searches of the unidentified taxon in Synergistaceae returned a 100% match to *Cloacibacillus porcorum* strain CL‐84, while the two unidentified Christensenellaceae and the Clostridiales paired only to uncultured bacterium.

### 3.4 Temporal variation of cloacal microbiota in captive crested iguanas postrelease

**FIGURE 4:**
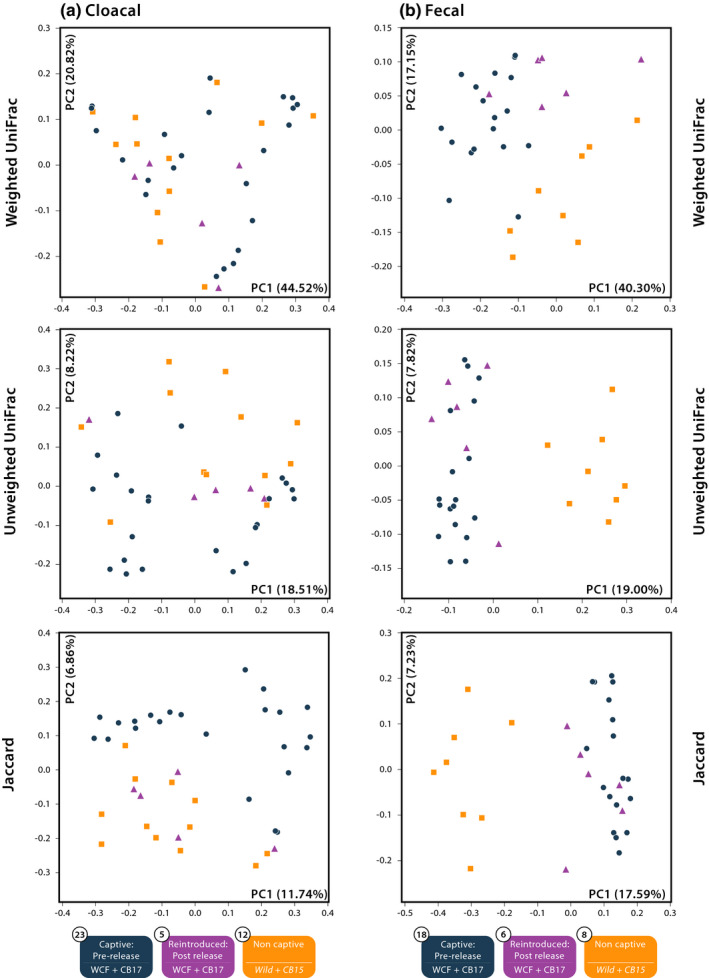
Principal coordinates analysis of reintroduced and initial (2017) (a) cloacal swabs and (b) fecal samples across three Fijian crested iguana treatment groups. Captive prerelease samples include wild‐caught founders (WCF) in captivity and captive‐born headstart individuals (CB2017) in captivity at Kula Eco Park collected February 2017. Noncaptive individuals consist of captive‐born individuals released onto Monuriki Island in 2015 (CB2015) and fully wild individuals on Monuriki Island (Wild). Postrelease treatments include formerly captive WCF and CB2017 individuals sampled in late April 2017, 2 months after release onto Monuriki Island

Comparisons of microbial communities from five cloacal samples taken shortly after host reintroduction against both captive and noncaptive microbial communities revealed no significant variation in alpha‐diversity metrics (Figure S6). Comparisons of reintroduced individuals with complete time‐series sampling yielded no significant difference in alpha‐diversity metrics pre‐ and postrelease. PCoA plots revealed limited clustering across all three conditions in weighted and unweighted UniFrac metrics though some grouping between reintroduced and noncaptive samples was apparent in Jaccard plots (Figure 4a). Plots of only individuals with complete time‐series sampling also showed inconsistent groupings (Figure S7). Adonis tests between reintroduced, captive, and noncaptive samples found significant differentiation in unweighted UniFrac (*R* ^2^ = 0.0883, *p* = 0.006) and Jaccard distances (*R* ^2^ = 0.0907, *p* = 0.001). Further pairwise comparisons between reintroduced samples and noncaptive samples uncovered no distinction in any beta metrics. Reintroduced samples were, however, significantly distinct from captive ones in the Jaccard metric (*R* ^2^ = 0.0601, *p* = 0.006). The average Jaccard distance among pairs of samples from reintroduced lizards was 0.83.

Microbial communities sourced from cloacal swabs in this subset were largely dominated by three phyla: Firmicutes, Proteobacteria, and Actinobacteria in all treatment groups. However, proportions of these taxa shifted between reintroduced, captive, and noncaptive conditions (Figure S8). We identified a single OTU that varied statistically between all three groups based on the Kruskal–Wallis tests, the unidentified taxon in Micrococcaceae matching *Nesterenkonia* sp. strain MadaFrogSkinBac.DB‐0.3605 (reintroduced mean reads = 0, captive = 19.1, noncaptive = 0). Interestingly, a number of OTUs that were commonly found in cloacal samples from captive animals including the additional strain of *Nesterenkonia*, the *Brevibacterium* sp., and the *Brachybacterium* sp. were nearly or entirely absent in rarefied reads of samples from reintroduced hosts (reintroduced mean reads = 0, 0.2, 0.2, respectively; Appendix S2).

### 3.5 Temporal variation of fecal microbiota in captive crested iguanas postrelease

**FIGURE 5:**
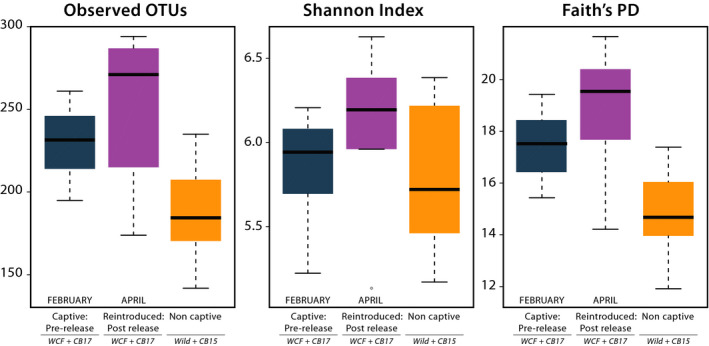
Alpha‐diversity metrics of fecal samples from reintroduced Fijian crested iguana hosts compared against initial samples. Captive prerelease samples include wild‐caught founders (WCF) in captivity and captive‐born headstart individuals (CB2017) in captivity at Kula Eco Park. Noncaptive individuals consist of captive‐born individuals released onto Monuriki Island in 2015 (CB2015) and fully wild individuals on Monuriki Island (Wild). Initial sample collection occurred in February 2017. Postrelease treatments include formerly captive WCF and CB2017 individuals sampled 2 months after release onto Monuriki Island in April 2017. Paired symbols denote significantly distinct treatment groups

We compared microbial communities in six fecal samples from reintroduced iguanas against those from captive and noncaptive samples and found significant differences in the number of observed OTUs (*p* < 0.001; reintroduced mean reads = 252, captive = 229, noncaptive = 188) and in Faith's PD (*p* < 0.001, reintroduced mean reads = 18.9, captive = 17.4, noncaptive = 14.9; Figure 5). Post hoc analyses of observed OTUs found significance only between reintroduced and noncaptive communities (*p* = 0.001). Faith's PD posthoc tests found significance between reintroduced and noncaptive communities (*p* = 0.003) as well as between captive and noncaptive communities (*p* = 0.003). Comparisons of reintroduced individuals with complete time‐series sampling yielded no significant difference in alpha‐diversity metrics pre‐ and postrelease. Plotted beta‐diversity metrics showed some clustering between treatment groups with reintroduced animals associating most closely with captive samples (Figure 4b). Significant differences in adonis tests were recorded in weighted UniFrac distances (*R* ^2^ = 0.3417, *p* = 0.001), unweighted UniFrac distances (*R* ^2^ = 0.2291, *p* = 0.001), and Jaccard distances (*R* ^2^ = 0.2197, *p* = 0.001) between reintroduced, captive, and noncaptive samples. Pairwise comparisons between reintroduced and captive samples were significantly distinct for all three metrics: weighted UniFrac (*R* ^2^ = 0.2493, *p* = 0.001), unweighted UniFrac (*R* ^2^ = 0.0899, *p* = 0.001), and Jaccard distances (*R* ^2^ = 0.0915, *p* = 0.001). Comparisons between reintroduced and noncaptive samples also produced significant differentiation in weighted UniFrac distances (*R* ^2^ = 0.3992, *p* = 0.006), unweighted UniFrac distances (*R* ^2^ = 0.3862, *p* = 0.002), and Jaccard distances (*R* ^2^ = 0.3747, *p* = 0.001). PCoA plots of individuals with complete time‐series sampling exclusively showed clustering with some overlap between groups (Figure S9). The average Jaccard distance among pairs of samples after release was 0.61.

Microbial communities found in fecal samples from reintroduced, captive, and noncaptive samples were primarily dominated by three phyla: Firmicutes, Bacteroidetes, and Proteobacteria. Relative abundances of these phyla varied between conditions (Figure S10). In comparing OTU relative abundances, Kruskal–Wallis tests retrieved nine OTUs that differed between all three treatments (Appendix S2). These included one *Acetobacterium* sp. (reintroduced = 3.2, captive = 82, noncaptive = 0), one *Akkermansia* sp. (reintroduced = 164.8, captive = 29.3, noncaptive = 0), one *Butyricimonas* sp. (reintroduced = 1.7, captive = 0, noncaptive = 0.3), one *Coprococcus* sp. (reintroduced = 2.5, captive = 0.3, noncaptive = 26.5), one *Ruminococcus* sp. (reintroduced = 7.7, captive = 95.3, noncaptive = 0), an unidentified Christensenellaceae (reintroduced = 121.7, captive = 53.4, noncaptive = 0), an unidentified Coriobacteriaceae (reintroduced mean reads = 0, captive = 0, noncaptive = 3.9), an unidentified Lachnospiraceae (reintroduced = 0.3, captive = 7.7, noncaptive = 0), and an unidentified Mogibacteriaceae (reintroduced = 0.5, captive = 0, noncaptive = 4.3). Scrutiny of additional taxa found in captive lizards yet absent from noncaptive ones yielded mixed results with some bacterial strains becoming more prevalent in reintroduced hosts and others becoming less prevalent (Appendix S2). The unknown Synergistaceae matching *C. porcorum,* for example, increased in mean relative abundance between conditions (reintroduced mean reads = 122.5, captive = 116.9) as did the noted *Bacteroides* sp. (reintroduced = 134.7, captive = 25.3). Meanwhile the second *Ruminococcus* sp. (reintroduced = 0.8, captive mean = 18.7), *Coprococcus* sp. (reintroduced = 0.7, captive mean = 11.0), and unidentified Clostridiales (reintroduced = 0.2, captive mean = 15.1) all decreased in relative abundances in reintroduced hosts (Appendix S2).

## 4 DISCUSSION

Our findings show that captive and noncaptive Fijian crested iguanas harbor distinct microbial communities regardless of sampling regime (cloacal versus fecal). These results expand on a growing body of evidence that suggests animals housed in captivity have distinct microbiomes when compared to wild conspecifics (Alfano et al., 2015; Cheng et al., 2015; Clayton et al., 2016; Eigeland et al., 2012; Jiang et al., 2017; Kohl et al., 2017; McKenzie et al., 2017; Ren et al., 2016; West et al., 2019; Zhu et al., 2011). In both cloacal and fecal sampling, captive (WCF and CB2017 grouped) and noncaptive (CB2017 and Wild grouped) iguanas harbored significantly different microbial communities in at least two beta‐diversity metrics (Figure 4). Further, Jaccard distances were consistently lower within captive treatments, suggesting a greater degree of shared OTU breadth and potentially homogenization among captive individuals. These findings are consistent with those seen in *Anolis sagrei* where alpha‐diversity measures were generally higher in captive animals compared with wild conspecific hosts, yet gut communities were more homogenous, and beta‐diversity metrics separated wild and captive hosts (Ren et al., 2016). In addition to harboring distinct microbial communities, a number of specific OTUs, particularly potential pathogens, were seen in greater abundances in captive over noncaptive Critically Endangered Fijian crested iguanas.

The introduction of potentially pathogenic bacteria has been documented previously in wild reptiles brought into temporary captivity (Jiang et al., 2017; Kohl et al., 2017) but not in a conservation initiative specifically designed to release captive animals into the wild. In cloacal samples from captive Fijian crested iguanas, one *Brachybacterium* sp., one *Brevibacterium* sp., and two *Nesterenkonia* spp. were present in rarefied reads while absent from noncaptive counterparts (Appendix S2). All three of these genera have species implicated as potential pathogens at least in humans (Gruner et al., 1993; Nakayama et al., 2009; Tamai et al., 2018). Fecal samples produced similar results where strains from multiple genera, including *Bacteroides*, *Cloacibacillus*, and *Ruminococcus* were found commonly in captive samples but absent in rarefied, noncaptive reads (Appendix S2). These three genera are also potentially pathogenic strains in humans (Domingo et al., 2015; Titécat et al., 2014; Wexler, 2007). Although determining the exact pathogenic capacities of particular microbes is outside the realm of this investigation, high abundances of potential pathogens in animals under human care support the possibility that headstart animals can harbor disease‐causing bacteria at significantly higher rates than animals living in the wild (Redford et al., 2012). Although microbial communities in hosts can shift rapidly on the scale of days to even hours in some cases (Costello et al., 2010; Ren et al., 2016), the impacts of releasing animals with elevated levels of what could be pathogenic microbiota have received little attention to date (Redford et al., 2012).

Reintroduction of captive Fijian crested iguanas into native habitats promoted restructuring of gut microbiomes toward noncaptive communities. After 2 months on Monuriki Island, cloacal samples from reintroduced iguanas appeared to harbor gut microbial communities more similar to noncaptive than to captive compositions (Figure 4 and S6). Additionally, noted potential pathogens in captive individuals were either absent or diminished in reintroduced hosts. Microbial assemblages generated from fecal samples, however, did not produce similar results. Instead, microbiota from fecal samples of reintroduced lizards seemingly resembled captive hosts more closely rather than noncaptive hosts (Figures 4 and 5). Potential pathogens also displayed differing trends with *Ruminococcus* spp. becoming less abundant in host iguanas two months after release and *Bacteroides* sp. and *Cloacibacillus* sp. becoming more abundant in samples taken from individuals after reintroduction. Such findings support previously proposed hypotheses that pathogens associated with human care may continue to impact headstart or reintroduced animals even after release (Bahrndorff et al., 2016; Redford et al., 2012; West et al., 2019). Despite fecal samples from reintroduced iguanas being significantly distinct from noncaptive samples, this differentiation does appear to be temporary. Released animals relocated onto Monuriki Island in 2015 (CB2015) contained gut microbial assemblages more closely associated with true wild iguanas rather than captive ones in both cloacal and fecal samples, suggesting that reacclimation of wild‐type microbiomes can occur after prolonged survival in native habitats (i.e., two years; Figure 2).

Although both cloacal and fecal sampling techniques recovered significant differentiation in gut microbial communities between captive and noncaptive Fijian crested iguanas (Figures 2 and 4), specific OTUs that varied between treatments were inconsistent. Further, differences were apparent in comparing assemblages from reintroduced lizards to those in captive and noncaptive hosts based on sampling regime (Figure 4). Cloacal samples from reptiles generally encapsulate the breadth of gut microbial diversity but vary significantly in abundances compared directly to hindgut samples while fecal samples tend to better represent gut diversity and abundances (Colston et al., 2015; Kohl et al., 2017). When assessing microbial communities in captive lizards for potential disease‐causing microbes, or in evaluating the restructuring of host microbiomes postrelease, multiple nonlethal gut microbial sampling techniques may be necessary to fully elucidate trends of interest.

Gut microbial communities in captive Fijian crested iguanas are distinct from those in noncaptive iguanas and this differentiation prevails for some time postrelease. However, the duration in which a host's microbial composition shifts to closely resemble true wild counterparts remains unclear. A continued need exists to monitor microbial communities in headstart animals postrelease to track animal well‐being (Bahrndorff et al., 2016; Jiménez & Sommer, 2017; Redford et al., 2012; West et al., 2019). Such studies could determine the influences of potential disease‐causing bacteria associated with captive upbringings on host survival, growth, and reproduction in the wild. Further, wild conspecifics in populations with introduced animals should be monitored closely for introduction of novel pathogens brought on from interaction with animals sourced from headstart programs (West et al., 2019). Such scenarios may justify the use of soft releases or probiotics prior to animal release to acclimatize gut microbiota in headstart individuals to natural conditions and eliminate possible disease‐causing agents before complete reintroduction to the wild (Redford et al., 2012; West et al., 2019). Along with increased monitoring of animal health, additional scrutiny of specific OTUs seen in differential abundances between headstart and wild animals that may be pathogenic is necessary to determine the virulence of such bacterial strains. Should these OTUs be minimally pathogenic, then no additional action may be necessary to mitigate their increased abundances while animals are in captive settings. Ultimately, consistent monitoring of hosts postrelease and further examination of possible pathogens are the next step toward improving our understanding of gut microbial ecology in endangered species with conservation significance.

## CONFLICT OF INTEREST

The authors declare no conflict of interest.

## AUTHOR CONTRIBUTIONS

**Samuel Joseph Eliades:** Formal analysis (equal); Funding acquisition (equal); Methodology (equal); Writing‐original draft (equal). **Joseph C. Brown:** Conceptualization (equal); Data curation; Funding acquisition; Methodology; Project administration (equal); Writing‐review & editing (equal). **Tim Colston:** Conceptualization (equal); Data curation; Formal analysis; Methodology; Writing‐review & editing (equal). **Robert N Fisher:** Conceptualization; Funding acquisition; Writing‐review & editing (equal). **Jone Niukula:** Data curation; Writing‐review & editing (equal). **Kim Gray:** Funding acquisition; Writing‐review & editing. **Jhabar Vadada:** Data curation; Writing‐review & editing (equal). **Sia Rasalato:** Data curation; Writing‐review & editing. **Cameron Siler:** Conceptualization (equal); Data curation (equal); Funding acquisition (equal); Project administration (equal); Writing‐review & editing (equal).

## Supporting information

Fig S1

Fig S2

Fig S3

Fig S4

Fig S5

Fig S6

Fig S7

Fig S8

Fig S9

Fig S10

Appendix S1

Appendix S2

## Data Availability

All 16S rRNA sequences have been deposited in the Sequence Read Archive (SRA) under accession no. PRJNA702127.
