# Supplementary figures and images for: Gut microbial ecology of the Critically Endangered Fijian crested iguana (Brachylophus vitiensis): Effects of captivity status and host reintroduction on endogenous microbiomes
Source: Ecol Evol. 2021 Mar 26;11(9):4731–43. doi: 10.1002/ece3.7373 (PMC8093715; doi:10.1002/ece3.7373)

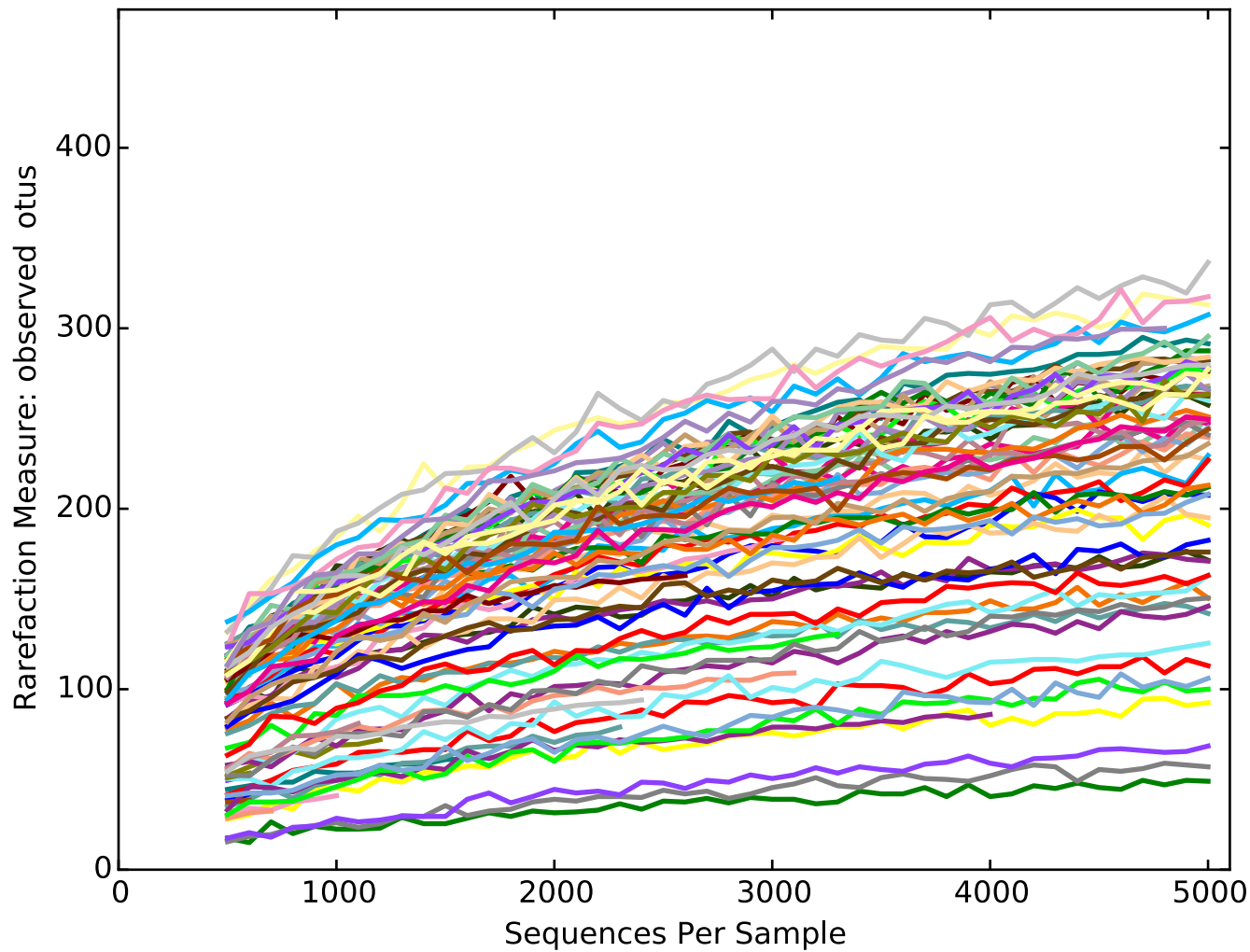

Supplement: Supplementary file 1 — Fig S1 [file ECE3-11-4731-s002.pdf]

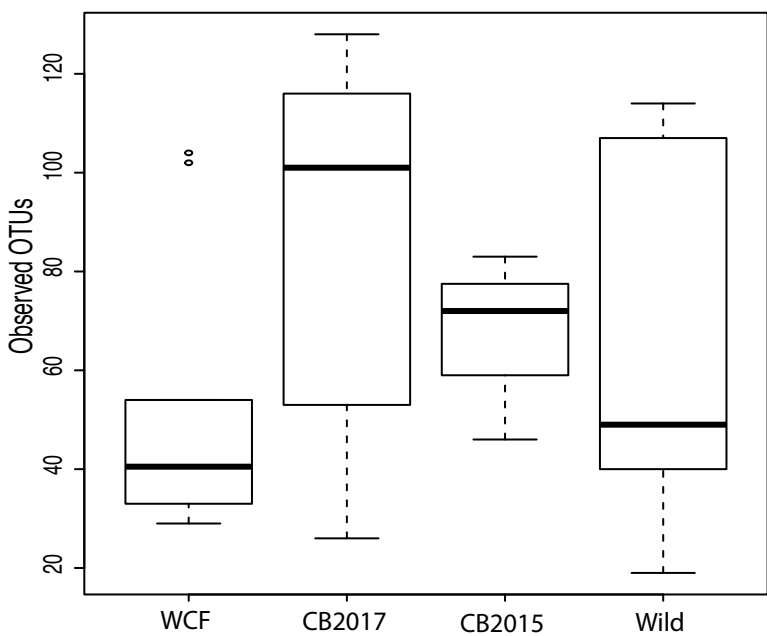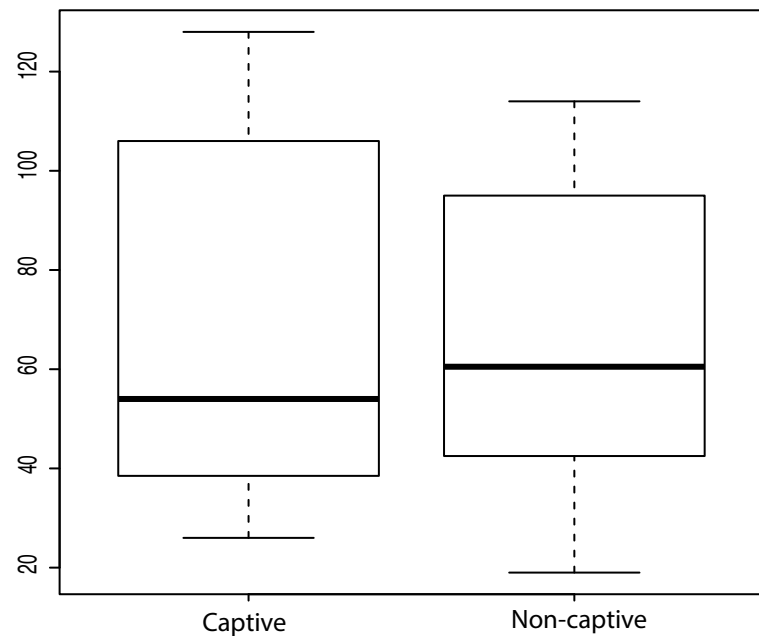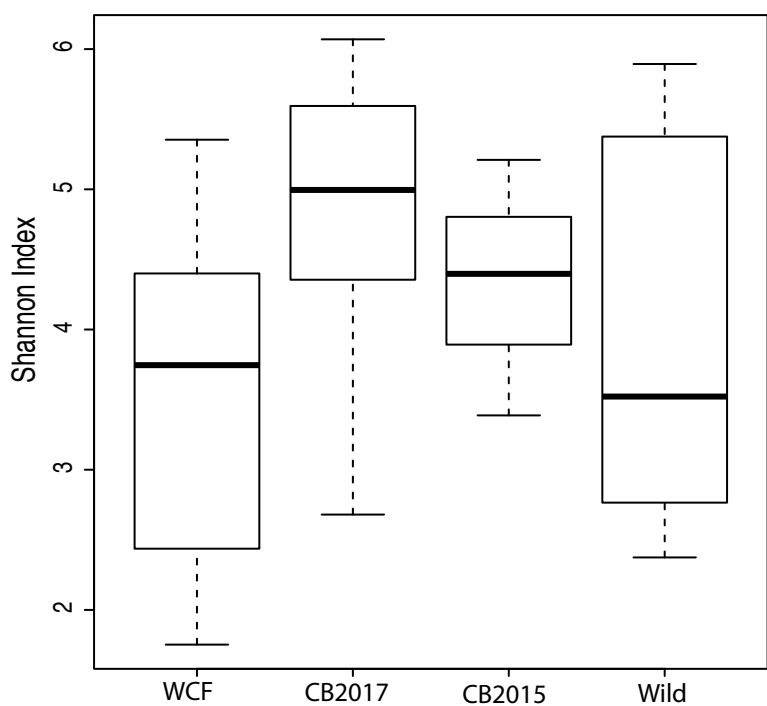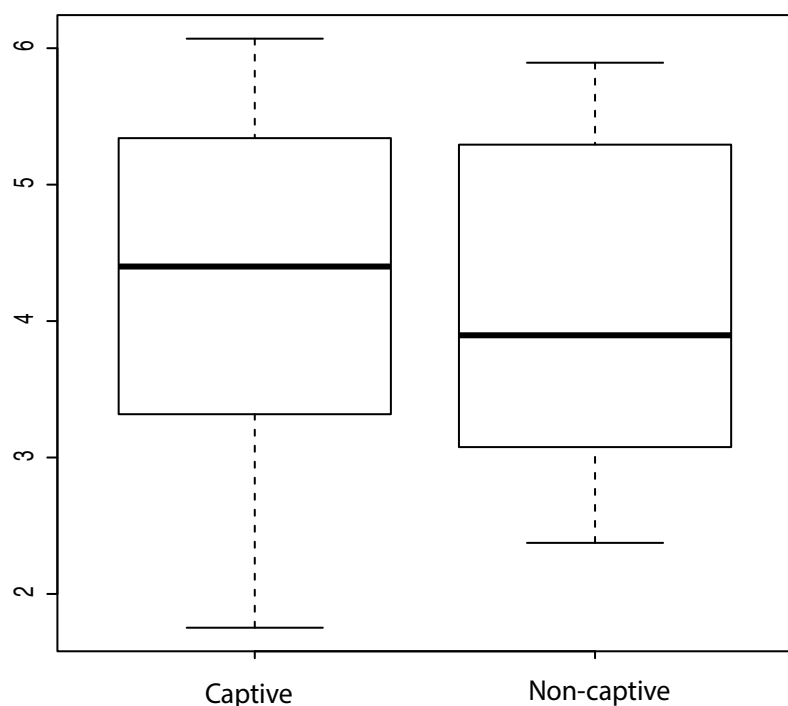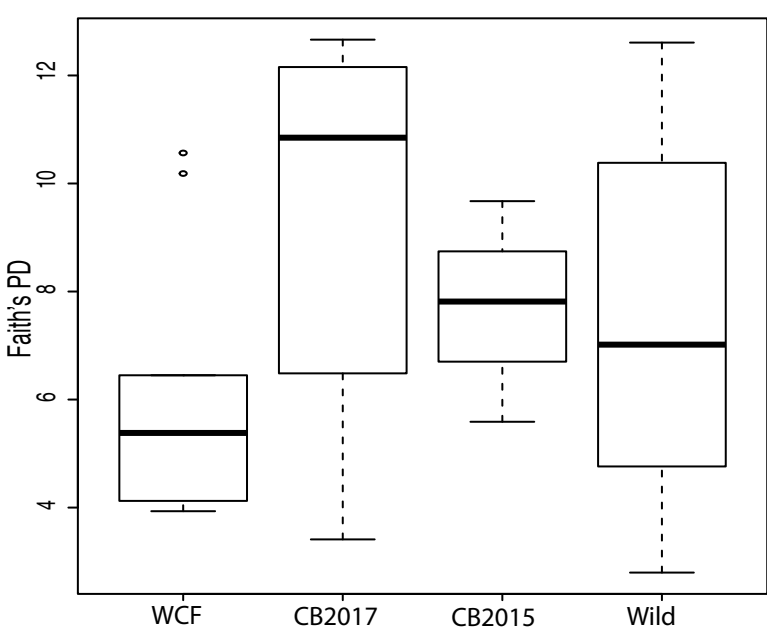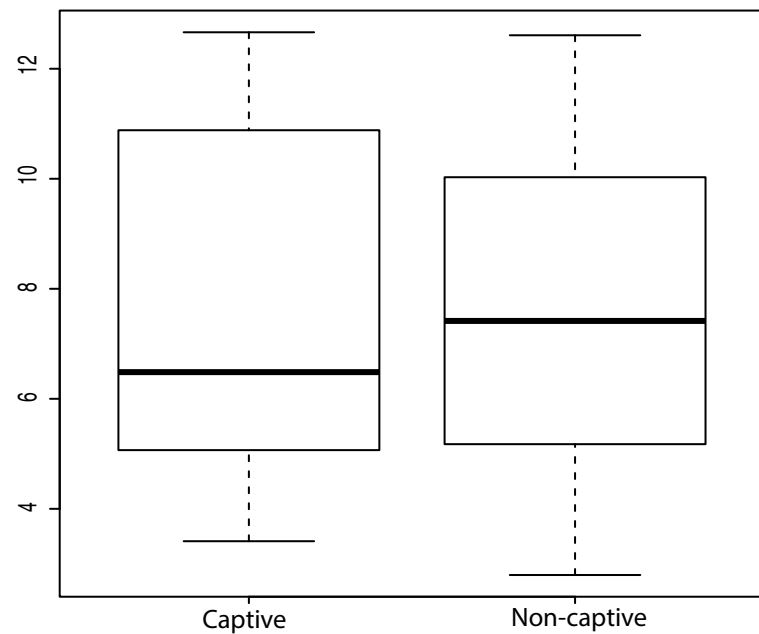

Supplement: Supplementary file 2 — Fig S2 [file ECE3-11-4731-s005.pdf]

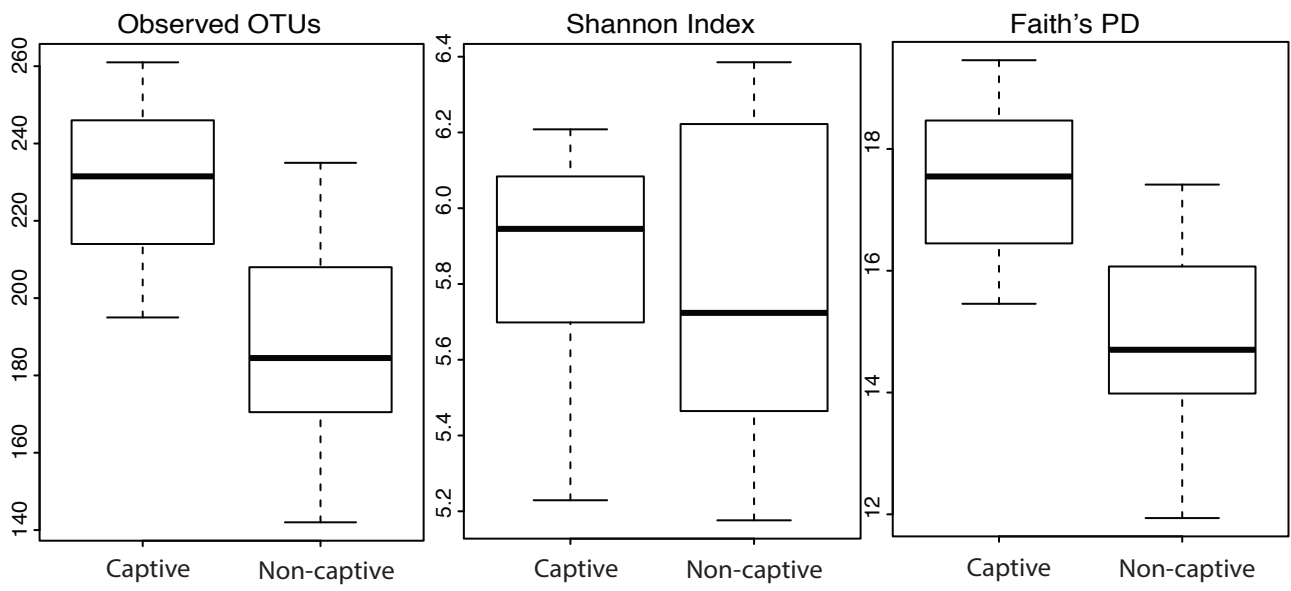

Supplement: Supplementary file 4 — Fig S4 [file ECE3-11-4731-s012.pdf]

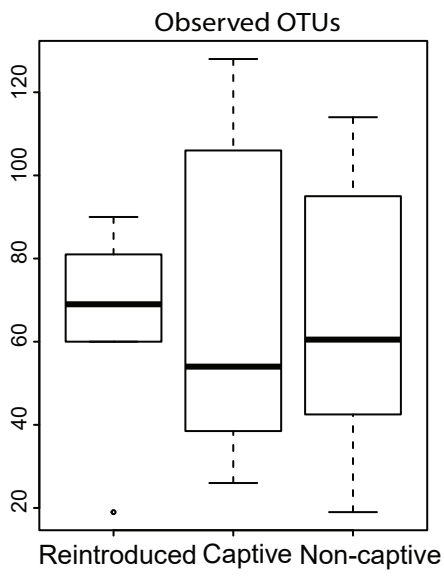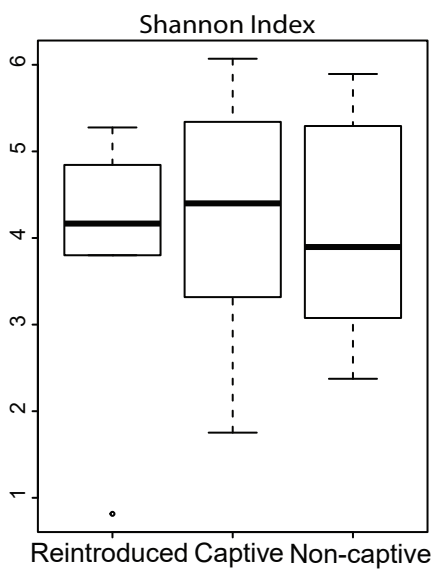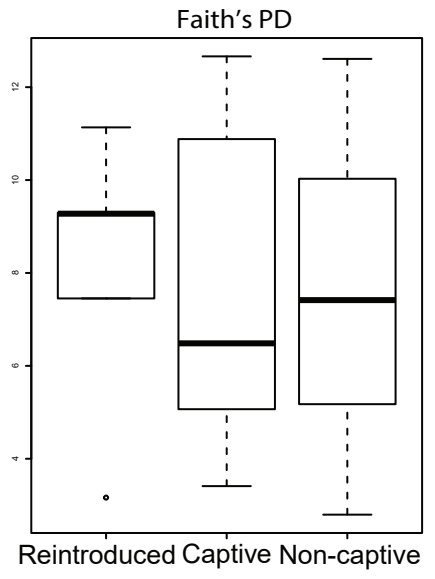

Supplement: Supplementary file 6 — Fig S6 [file ECE3-11-4731-s004.pdf]

## Cloacal

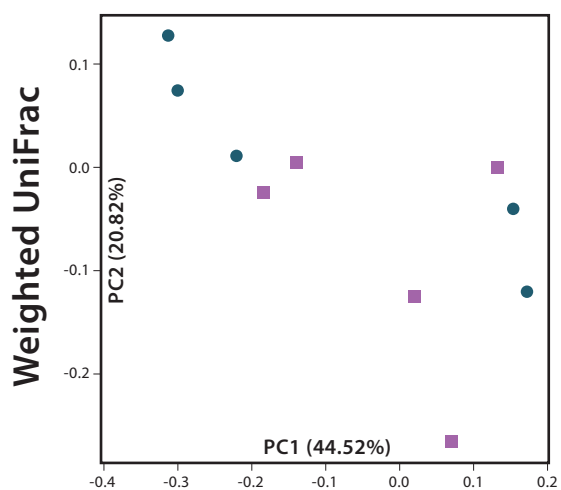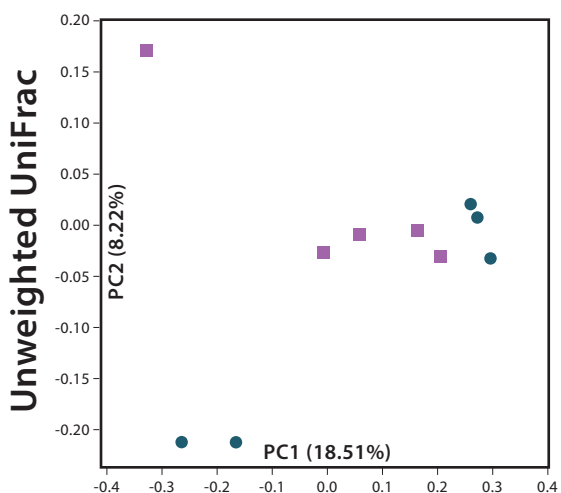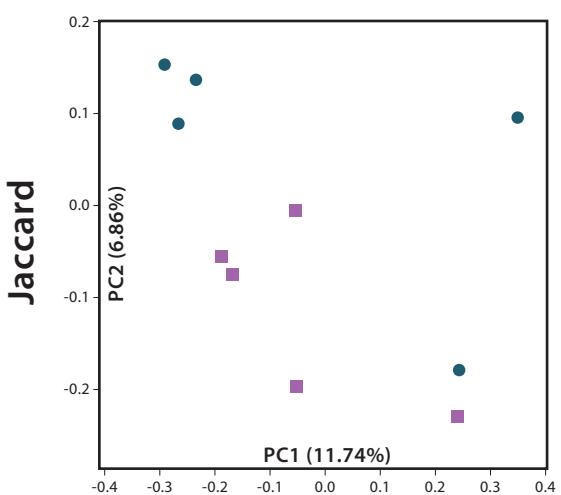

23

Captive:  
Pre-release  
WCF + CB17

5

Reintroduced:  
Post-release  
WCF + CB17

Supplement: Supplementary file 7 — Fig S7 [file ECE3-11-4731-s001.pdf]

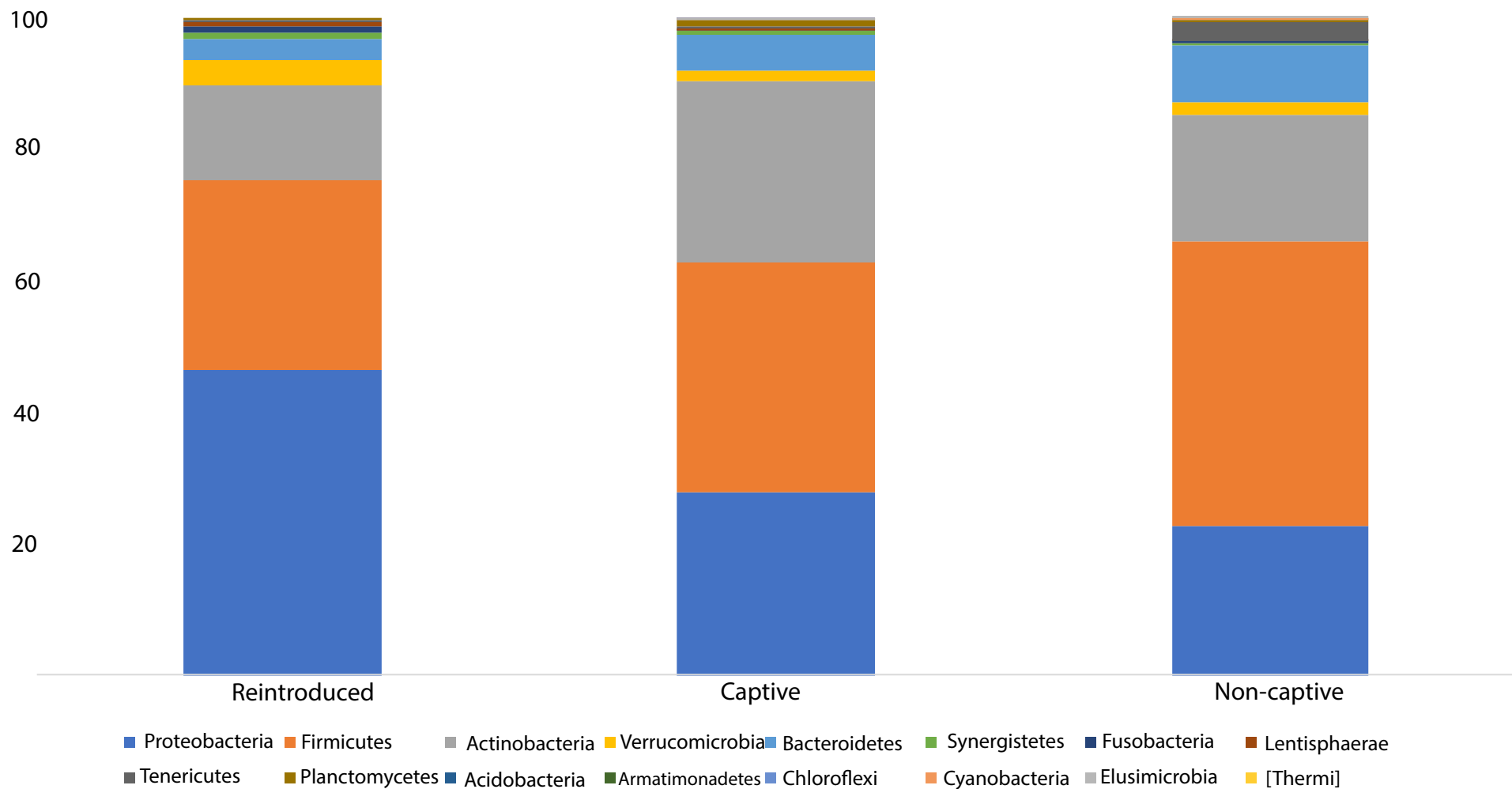

Supplement: Supplementary file 8 — Fig S8 [file ECE3-11-4731-s007.pdf]

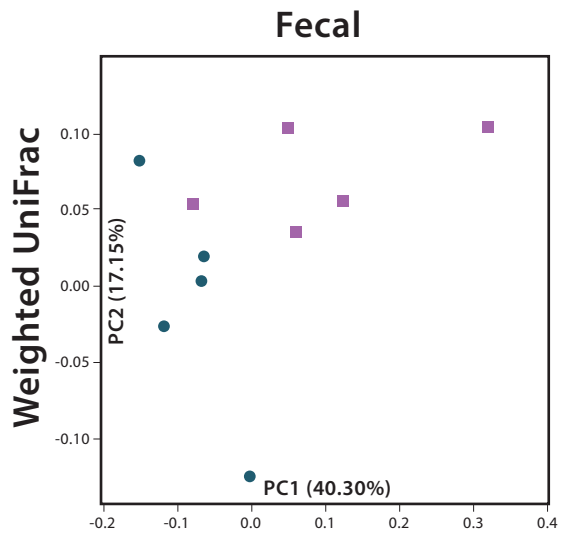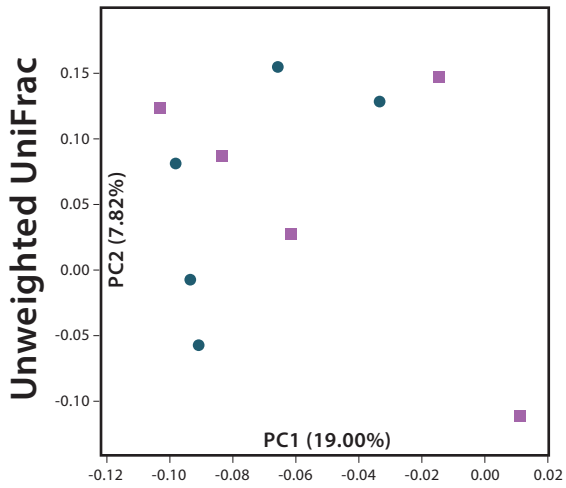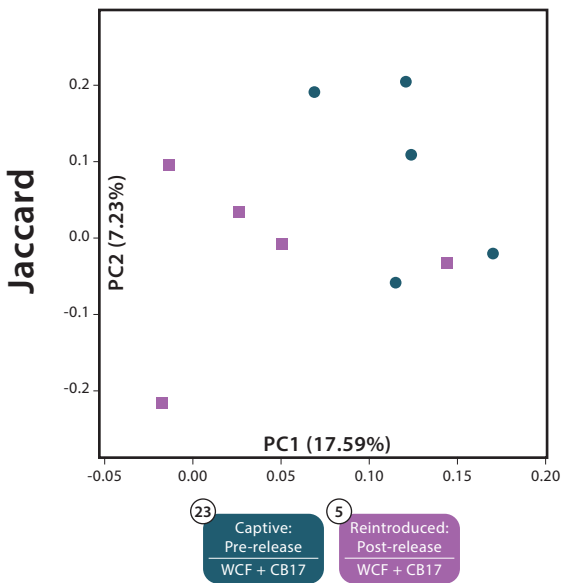

Supplement: Supplementary file 9 — Fig S9 [file ECE3-11-4731-s011.pdf]

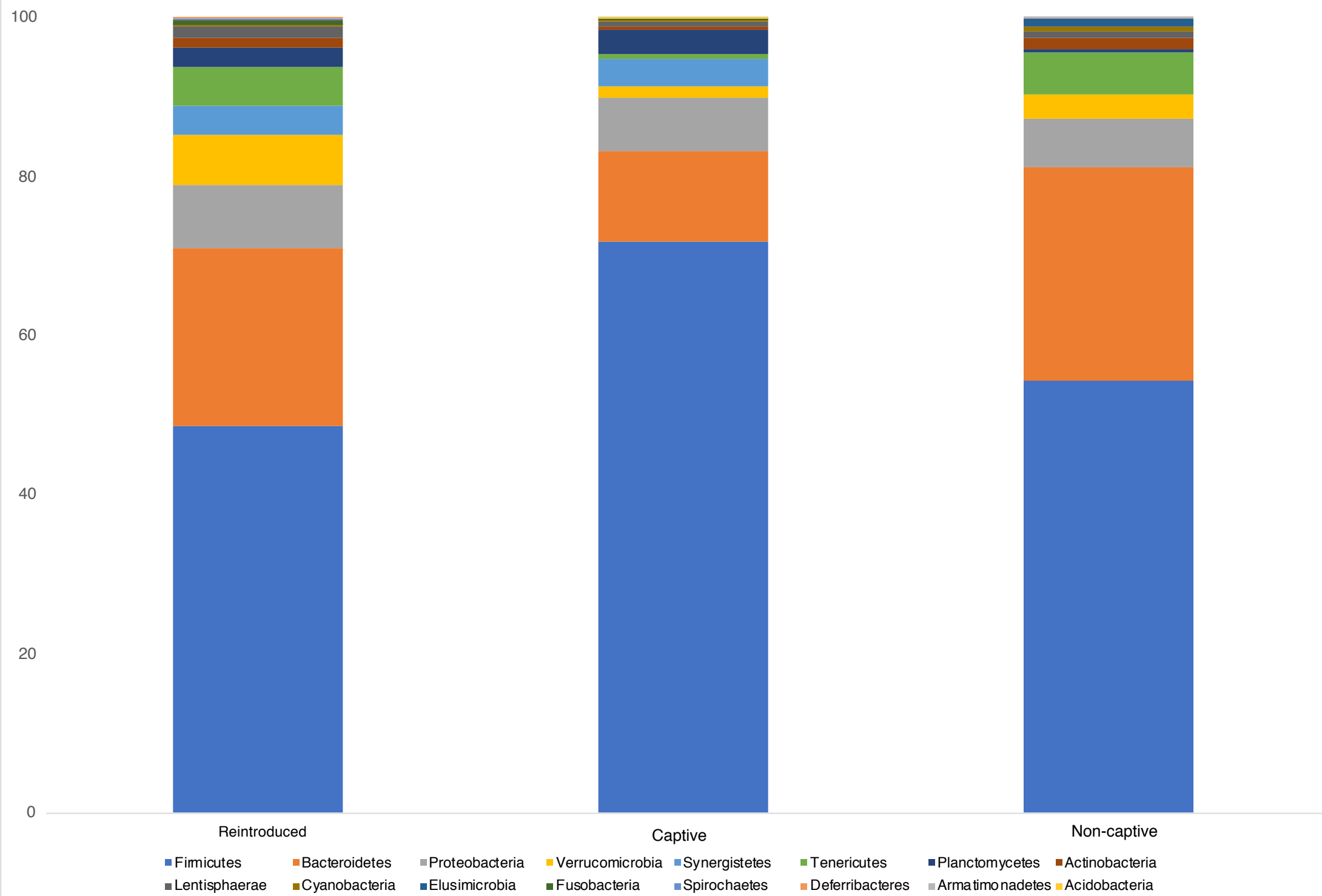

Supplement: Supplementary file 10 — Fig S10 [file ECE3-11-4731-s006.pdf]
