# Supplementary material for: Gut microbial ecology of the Critically Endangered Fijian crested iguana (Brachylophus vitiensis): Effects of captivity status and host reintroduction on endogenous microbiomes
Source: Ecol Evol. 2021 Mar 26;11(9):4731–43. doi: 10.1002/ece3.7373 (PMC8093715; doi:10.1002/ece3.7373)
Supplement: Supplementary file 3 — Fig S3 [file ECE3-11-4731-s003.pdf]

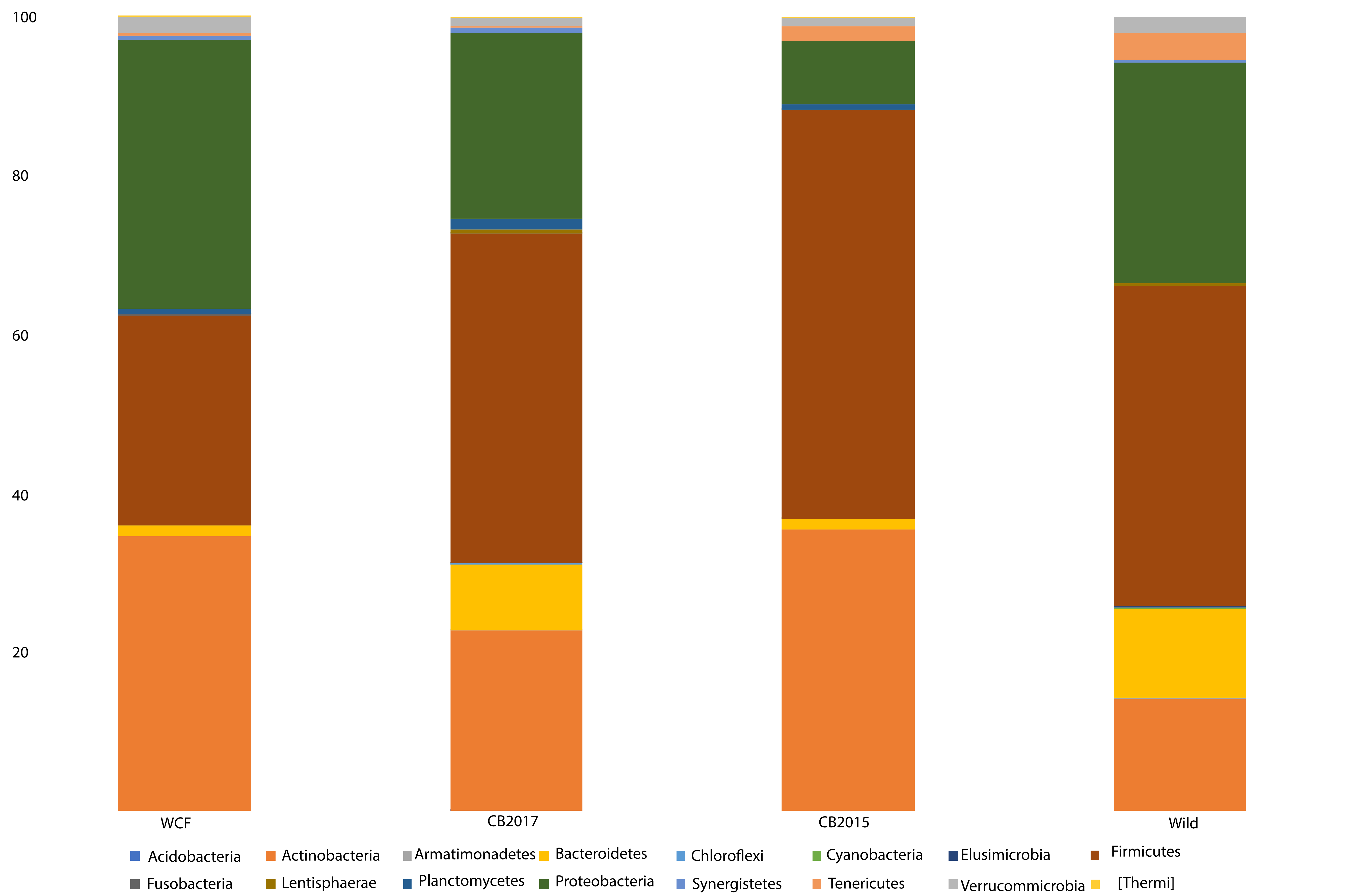
100
80
60
40
20
WCF
CB2017
CB2015
Wild
Acidobacteria
Actinobacteria
Armatimonadetes
Bacteroidetes
Chloroflexi
Cyanobacteria
Elusimicrobia
Firmicutes
Fusobacteria
Lentisphaerae
Planctomycetes
Proteobacteria
Synergistetes
Tenericutes
Verrucommicrobia
[Thermi]
