# Supplementary material for: Gut microbial ecology of the Critically Endangered Fijian crested iguana (Brachylophus vitiensis): Effects of captivity status and host reintroduction on endogenous microbiomes
Source: Ecol Evol. 2021 Mar 26;11(9):4731–43. doi: 10.1002/ece3.7373 (PMC8093715; doi:10.1002/ece3.7373)
Supplement: Supplementary file 5 — Fig S5 [file ECE3-11-4731-s009.pdf]

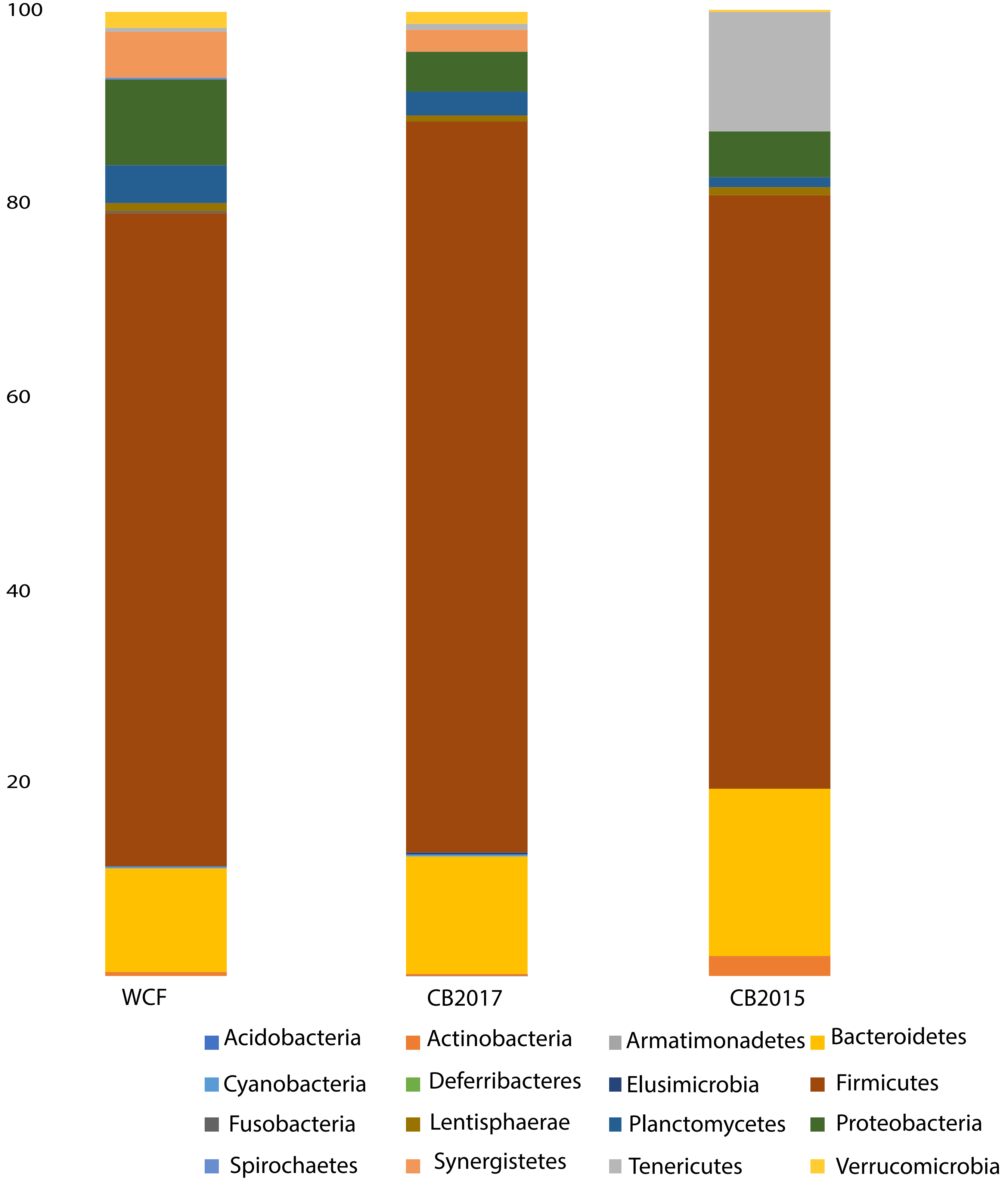
100
80
60
40
20
WCF
CB2017
CB2015
Wild
Acidobacteria
Actinobacteria
Armatimonadetes
Bacteroidetes
Cyanobacteria
Deferribacteres
Elusimicrobia
Firmicutes
Fusobacteria
Lentisphaerae
Planctomycetes
Proteobacteria
Spirochaetes
Synergistetes
Tenericutes
Verrucomicrobia
